# Catatonia knowledge in medical practitioners from low-resource settings

**DOI:** 10.4102/sajpsychiatry.v32i0.2676

**Published:** 2026-10-07

**Authors:** Andrea K. Kaddu, Daisy C. Saina, Zahra Morawej, Wilber Ssembajjwe, Andrew S. Ssemata, Rossette I. Ndigamanya, Kristien van der Walt, Allan Kalungi, Dickens Akena, Noeline Nakasujja, Emmanuel K. Mwesiga

**Affiliations:** 1 Department of Psychiatry, School of Medicine, Makerere University College of Health Sciences, Kampala, Uganda; 2 Department of Psychiatry, Faculty of Medicine, Moi University, Eldoret, Kenya; 3 Department of Psychiatry and Behavioural Sciences, School of Medicine, Kairuki University, Dar es Salaam, Tanzania; 4 Medical Research Council/Uganda Virus Research Institute, London School of Hygiene and Tropical Medicine, Entebbe, Uganda; 5 Department of Psychiatry and Mental Health, University of Cape Town, Cape Town, South Africa

**Keywords:** catatonia, knowledge, medical practitioners, low-resource settings, African Catatonia Registry

## Abstract

**Background:**

Catatonia is a neuropsychiatric emergency that requires rapid intervention to prevent complications. It is unclear whether African medical practitioners have the requisite knowledge to identify and manage catatonia.

**Aim:**

To examine knowledge of catatonia among medical practitioners across partnering institutions of the African Catatonia Registry (ACR), a multi-country resource for research in Africa.

**Setting:**

The study was conducted among medical practitioners across the ACR partnering institutions.

**Methods:**

The study was a descriptive, cross-sectional online survey comprising 63 questions to examine knowledge of catatonia. Responses were scored with a positive mark if correct and a negative mark if wrong, then standardised and dichotomised to indicate low or sufficient knowledge.

**Results:**

Out of 321 participants, nearly half (46.7%) were psychiatrists, and 50.8% had sufficient knowledge of catatonia. Multivariate analysis showed that knowledge varied by sub-domain, country, specialty and prior experience with catatonia. Specialisation in psychiatry was significantly associated with sufficient knowledge of classification (adjusted risk ratio [aRR] 2.15 [95% confidence intervals {CI}: 1.35–3.43]), diagnostic symptoms (aRR = 3.30 [95% CI: 1.62–6.69]), screening (aRR = 4.72 [95% CI: 2.25–9.91]) and rating scales (aRR = 3.34 [95% CI: 1.32–8.46]). Prior experience with catatonia was also associated with sufficient knowledge across these areas (all *p* ≤ 0.05).

**Conclusion:**

While knowledge of catatonia and its diagnostic criteria is sufficient among practitioners, knowledge of its symptoms, treatment and complication management remains low.

**Contribution:**

The study highlights the need for the ACR to promote improvements in the identification and management of catatonia to achieve better patient outcomes.

## Introduction

Catatonia is a neuropsychiatric emergency that requires rapid intervention to prevent serious complications, including mortality. First described by Kalbhaum in 1874, catatonia may present with decreased, increased or abnormal psychomotor activity (catalepsy, waxy flexibility, stupor and posturing), affective signs (agitation) and cognitive-behavioural signs (mutism, negativism, mannerisms, stereotypies, grimacing, echolalia and echopraxia).^1,2^ Catatonia presents with varying prevalence across populations, including medical, substance use and psychiatric units, and across different age groups and in hospital and community settings.^3,4^ A comprehensive meta-analysis, including 74 studies and 107 304 individuals from 99 independent samples across all continents, showed that the overall pooled mean prevalence of catatonia was 9.2% among subjects diagnosed with a variety of psychiatric or medical conditions.^5^ Few studies on catatonia have been conducted in Africa, with available evidence estimating its prevalence at approximately 5.9%.^5^ A recent study in South Africa reports a prevalence rate of 18.3% within an acute mental health unit.^6^ Prompt identification and treatment are aided by various diagnostic criteria, including the ICD-11 and DSM-5, as well as screening and diagnostic instruments like the Bush Francis Catatonia Rating scale.^7^ The management of catatonia involves treatment with benzodiazepines or electroconvulsive therapy, as well as treatment of the underlying condition. Prompt recognition and appropriate management are required to prevent complications like malnutrition, dehydration, infections, decubitus ulcers, contractures, deep vein thrombosis and pulmonary embolism.^6,8,9,10^ Clinicians require adequate knowledge of catatonia to enable timely identification and appropriate treatment, which are essential to preventing associated morbidity and mortality.

Several recent studies indicate substantial gaps in clinicians’ knowledge on catatonia, particularly in recognising its clinical features.^11,12^ In low-resource settings, particularly in Africa, no studies have been conducted on the knowledge of catatonia among health practitioners. However, evidence from other studies on catatonia shows recurring gaps in knowledge, reflected in under-recognition and misdiagnosis.^13^ It remains unclear whether medical practitioners from low-resource settings often see patients with catatonia and can correctly identify symptoms, make correct diagnoses using various diagnostic criteria and assess severity using different rating scales. Further, it is important to examine medical practitioners’ knowledge of catatonia management to prevent complications. Lastly, since catatonia is present across populations, it would be important to examine whether this knowledge differs across specialities and locations. This is important because non-specialist providers and health workers play an important role in the management of psychiatric disorders in low-resource settings.^14^

The African Catatonia Registry (ACR) aims to improve catatonia research in participating academic institutions in Ethiopia (Addis Ababa University), Kenya (Moi University), South Africa (University of Cape Town), Tanzania (University of Dodoma) and Uganda (Makerere University). Initial approvals have been obtained to develop a registry of catatonia patients in these countries to further research into catatonia. In this study, we aimed to examine knowledge of catatonia across partnering institutions. This knowledge is important for the ACR to identify gaps in the identification and management of catatonia and to develop training programmes.

## Research methods and design

### Study design

A descriptive cross-sectional study using an online survey between 08 January 2024 and 25 March 2024.

### Study population

The study population comprised medical practitioners from the partnering institutions, regardless of cadre or speciality. We excluded undergraduate medical students from this study as they are not expected to identify patients for the registry.

### Sampling procedure

Purposive sampling was used, with the survey link shared across various fora within the partnering institutions. A formal sample size calculation was performed *a priori* to determine the minimum number of participants required to support the planned inferential analyses.

### Study variables

This survey was developed at Moi Teaching and Referral Hospital, the Medical Research Council of Uganda and Makerere University and comprised 63 structured questions to examine knowledge of catatonia identification, treatment and management of complications. The questionnaire included questions on the definition of catatonia, causes, classification, symptoms, screening, diagnostic symptoms, rating scales, treatment and complications. It also included questions about whether the practitioner had ever seen or treated catatonia and whether they had seen or treated it in the previous year.

### Study procedure

The link to the document was circulated to the various institutions using WhatsApp and email. Participant contact details were not obtained from any private or restricted source. Recruitment invitations were circulated through existing departmental and institutional communication platforms (departmental WhatsApp groups and official mailing lists) to which members had already voluntarily subscribed. No contact list was extracted, stored or reused by the research team, and participation was entirely voluntary and self-initiated by clicking the survey link. The informed consent statement was presented at the beginning of the document, and participants who consented could proceed to the questionnaire. Participants who did not give informed consent automatically reached the end of the form, where they could submit without completing the questionnaire. Each participant could complete the questionnaire only once.

### Data management and analysis

Statistical analyses were conducted using Stata release 18. All categorical variables were summarised using frequencies and proportions. Catatonia knowledge was assessed using 63 structured questions with yes, no, or don’t know responses. For every correct response, the participant was awarded a mark, and a negative mark was attached to a wrong answer. Responses with ‘don’t know’ were scored with zero. The scores were then standardised using Equation 1:

$$Standard\;score=\frac{Row\;score-Mean\;score}{Standard\;deviation}$$ [Eqn 1]

The standard score was then dichotomised: those with negative scores were considered to have low knowledge, whereas those with positive scores were considered to have sufficient knowledge about catatonia.

The chi-square test for independence at an alpha level of 0.05 was then used to assess the relationship between socio-demographic characteristics and knowledge of catatonia.

Catatonia knowledge was assessed using the same standardisation procedure across the following domains: classification, symptoms, screening, diagnostic symptoms, treatment and complications. For each domain, we constructed a binary outcome. Bivariate modified Poisson regression analyses were performed for each predictor variable, with sufficient knowledge as the outcome, assessing crude associations between each outcome and each explanatory variable.

In the multivariate modified Poisson regression models, all selected variables were included simultaneously, allowing estimation of adjusted risk ratios (aRR) with 95% confidence intervals (CI) for each factor. The Hosmer–Lemeshow test was used to assess goodness-of-fit, and the area under the ROC curve (AUC) was calculated to evaluate the model’s ability to discriminate between those with low and sufficient knowledge. Multicollinearity was assessed using the variance inflation factor (VIF), and residuals were examined for outliers or influential data points that might skew the results.

The final model was selected based on the lowest Akaike Information Criterion (AIC) and a non-significant Hosmer–Lemeshow test result, indicating a good fit. The results from this model are presented as aRR with 95% CIs.

### Data safety

All data collected were handled confidentially and used solely for research purposes. The handling and storage of data complied with approved ethical guidelines to ensure participant privacy and data integrity throughout the study period.

### Ethical considerations

Ethical approval for the study was obtained from the Institutional Research and Ethics Committee of Moi University (MTRH/MU-IREC). The approval number is FAN: 0004596. The socio-demographic questions did not collect the names or hospital of the health worker, so they could not be identified. Participants were not reimbursed for their time.

## Results

### Participants

A total of 321 participants from Ethiopia, Kenya, Tanzania and Uganda completed the survey. The majority of participants were Ugandans (*n* = 139, 43.3%), aged 26–35 years (*n* = 181, 56.4%), and most were academicians or clinicians (*n* = 158, 48.0%). Nearly half were psychiatrists (*n* = 150, 46.7%), and the highest level of education for most was a bachelor’s degree (*n* = 144, 44.9%). The socio-demographics are summarised in Table 1.

**TABLE 1:** Socio-demographic characteristics of the participants (*N* = 321).

Factor	Level	Frequency	%
Gender	Male	182	56.7
	Female	139	43.3
Age (years)	≤ 25	25	7.8
	26–35	181	56.4
	36–45	73	22.7
	46–55	24	7.5
	> 55	08	2.5
Country	Uganda	139	43.3
	Kenya	30	9.5
	Tanzania	75	23.4
	Ethiopia	57	17.8
	Other†	20	6.2
Occupation	Academician and/or Clinician	154	48.0
	Registrar or SHO	86	26.8
	Graduate student	19	5.9
	Other	57	17.8
Specialty	Medicine	35	10.9
	Surgery	14	4.4
	Obstetrics	09	2.8
	Paediatrics	09	2.8
	Psychiatry	150	46.7
	Other‡	89	27.7
Sub-specialty	Neurology (Adult and Paediatric)	04	1.2
	Neurosurgery (Adult and Paediatric)	02	0.6
	General psychiatry	91	28.4
	Child and adolescent psychiatry	07	2.1
	Addiction psychiatry	04	1.2
	Other psychiatry	17	5.3
	Infectious disease (Adult and Paediatric)	16	5.0
	Other§	104	32.4
Highest level of education	Bachelors	144	44.9
	MSc/Masters	127	39.6
	PhD	07	2.2
	Others¶	37	11.5

SHO, Senior House Officer; PhD, Doctor of Philosophy; MSc, Master of Science.

† , Other countries include: South Africa, the Democratic Republic of Congo and Somalia;

‡ , Other specialities: medical officers and doctors in early training, such as interns;

§ , Other subspecialties: cardiology, endocrinology, nephrology and emergency medicine;

¶ , Other highest level of education: diploma-level holders, certificate in nursing.

### Knowledge and clinical experience with catatonia

There were almost similar numbers of participants with low (*n* = 158, 49.2%) and sufficient (*n* = 168, 50.8%) catatonia knowledge, with no statistically significant difference between the two groups. However, statistical differences were present when these categories were compared across countries, specialities and sub-specialities. The majority of participants (*n* = 268, 83.5%) had seen a patient with catatonia before, although fewer (*n* = 174, 54.2%) had seen one within the last year. Some participants (*n* = 216, 67.3%) had also ever treated a patient with catatonia, but fewer (*n* = 150, 46.7%) had done so in the last year. The differences in knowledge levels between participants who had ever seen or treated a patient with catatonia and those who had seen or treated a patient within the last year were significant (*p* < 0.001). Other results are shown in Table 2.

**TABLE 2:** Knowledge, clinical experience with catatonia and associated factors.

Factor	Level	Catatonia knowledge				*p*-value
		Low (*N* = 158, 49.2%)		Sufficient (*N* = 163, 50.8%)		
		*n*	%	*n*	%	
Gender	-	-	-	-	-	0.058
	Male	98	62.0	84	51.5	-
	Female	60	38.0	79	48.5	-
Age (years)	-	-	-	-	-	0.304
	25 & below	17	11.0	8	5.1	-
	26–35	86	55.8	95	50.5	-
	36–45	36	23.4	37	23.6	-
	46–55	10	6.5	14	8.9	-
	Above 55	5	3.3	3	1.9	-
Country	-	-	-	-	-	< 0.001
	Uganda	83	52.5	56	34.4	-
	Kenya	11	7.0	19	11.7	-
	Tanzania	44	27.9	31	19.0	-
	Ethiopia	17	10.8	40	24.5	-
	Other†	3	1.9	17	10.4	-
Speciality	-	-	-	-	-	< 0.001
	Medicine	29	19.5	6	3.8	-
	Surgery	9	6.0	5	3.2	-
	Obstetrics	6	4.0	3	1.9	-
	Paediatrics	7	4.7	2	1.3	-
	Psychiatry	40	26.9	110	70.1	-
	Other‡	58	38.9	31	19.8	-
Sub-speciality	-	-	-	-	-	< 0.001
	Neurology	3	2.5	1	0.8	-
	Neurosurgery	-	-	2	1.6	-
	General psychiatry	26	21.5	65	53.3	-
	Child and adolescent psychiatry	2	1.7	5	4.1	-
	Addiction psychiatry	2	1.7	2	1.6	-
	Other psychiatry	5	4.1	12	9.8	-
	Infectious disease	12	9.9	2	1.6	-
	Other§	71	58.7	33	27.1	-
Have you ever seen a patient with catatonia?	-	-	-	-	-	< 0.001
	No	47	29.8	6	3.7	-
	Yes	111	70.3	157	96.3	-
Have you seen a patient within the last year?	-	-	-	-	-	< 0.001
	No	96	60.8	51	31.3	-
	Yes	62	39.2	112	68.7	-
Have you ever treated a patient with catatonia?	-	-	-	-	-	< 0.001
	No	80	50.6	25	15.3	-
	Yes	78	49.4	138	84.7	-
Have you treated catatonia in the last year?	-	-	-	-	-	< 0.001
	No	107	67.7	64	39.3	-
	Yes	51	32.3	99	60.7	-

† , Other countries include: South Africa, the Democratic Republic of Congo and Somalia;

‡ , Other specialities include: medical officers and doctors in early training, such as interns;

§ , Other sub-specialities include: cardiology, endocrinology, nephrology and emergency medicine.

### Catatonia knowledge according to the sub-domains

There were 196 (61.1%) participants who had sufficient knowledge of catatonia classification systems. However, few participants had sufficient knowledge of catatonia symptoms (*n* = 103, 32.1%), catatonia rating scales (*n* = 101, 31.5%), treatment (*n* = 139, 43.3%) and complications (*n* = 132, 41.1%). Other proportions of knowledge on catatonia screening, catatonia rating scales, treatment and complications are shown in Table 3.

**TABLE 3:** Catatonia knowledge according to the sub-domains.

Domain	Level	Frequency	%
Catatonia classification	Low knowledge	125	38.9
	Sufficient knowledge	196	61.1
Catatonia symptoms	Low knowledge	218	67.9
	Sufficient knowledge	103	32.1
Diagnostic symptoms	Low knowledge	158	49.2
	Sufficient knowledge	163	50.8
Screening	Low knowledge	164	51.1
	Sufficient knowledge	157	49.9
Catatonia rating scales	Low knowledge	220	68.5
	Sufficient knowledge	101	31.5
Treatment	Low knowledge	182	56.7
	Sufficient knowledge	139	43.3
Complications	Low knowledge	189	58.9
	Sufficient knowledge	132	41.1

### Factors associated with catatonia knowledge according to the sub-domains at multivariate analysis

#### Catatonia classification

The country of practice was strongly associated with classification accuracy. Compared with Uganda, participants from Kenya (aRR = 1.35; 95% CI: 1.05–1.73) and those from the other category (aRR = 1.54; 95% CI: 1.25–1.90) demonstrated significantly more sufficient classification knowledge (*p* < 0.001). Speciality also predicted sufficient knowledge of classification. Compared to medicine, participants from paediatrics, psychiatry and other specialities performed better, with psychiatry having the strongest association (aRR = 2.15; 95% CI: 1.35–3.43; *p* < 0.001). Subspecialty was also significantly associated with knowledge of classification. Compared to neurology, all other subspecialties demonstrated lower knowledge for catatonia classification (*p* = 0.007). Prior experience with catatonia significantly improved classification knowledge. This included having ever seen a patient (aRR = 2.36; 95% CI: 1.51–3.68; *p* < 0.001), seen a patient in the past year (aRR = 1.50; 95% CI: 1.23–1.83; *p* < 0.001), ever treated a patient (aRR = 1.67; 95% CI: 1.29–2.17; *p* < 0.001) and treated a patient in the past year (aRR = 1.50; 95% CI: 1.24–1.82; *p* < 0.001).

#### Diagnostic symptoms

The country of practice was significantly associated with knowledge of diagnostic symptoms. Participants from Kenya (aRR = 1.22; 95% CI: 0.83–1.79), Ethiopia (aRR = 1.39; 95% CI: 1.05–1.85) and the other category (aRR = 1.77; 95% CI: 1.33–2.35) performed better than those from Uganda (*p* < 0.001). The speciality was also significant. Compared with medicine, both psychiatry (aRR = 3.30; 95% CI: 1.62–6.69) and other specialities (aRR = 2.34; 95% CI: 1.14–4.83) showed more sufficient knowledge of diagnostic symptoms (*p* = 0.005). Prior experience with catatonia was associated with sufficient knowledge of diagnostic symptoms. This included having ever seen a patient (aRR = 2.01; 95% CI: 1.23–3.26; *p* = 0.005), having seen one in the past year (aRR = 1.64; 95% CI: 1.27–2.11; *p* < 0.001), having ever treated a patient (aRR = 1.85; 95% CI: 1.33–2.57; *p* < 0.001) and treating in the past year (aRR = 1.42; 95% CI: 1.11–1.81; *p* = 0.004).

#### Catatonia screening

The country of practice was strongly associated with knowledge of catatonia screening. Participants from Ethiopia (aRR = 2.13; 95% CI: 1.64–2.76) and the other countries category (aRR = 1.85; 95% CI: 1.31–2.61) performed significantly better than those from Uganda (*p* < 0.001).

Speciality was significant, with psychiatry showing dramatically more sufficient knowledge compared with medicine (aRR = 4.72; 95% CI: 2.25–9.91; *p* < 0.001). Experience with catatonia improved knowledge of screening. This included having ever seen a patient (aRR = 2.96; 95% CI: 1.52–5.75; *p* = 0.001), seen one in the last year (aRR = 2.55; 95% CI: 1.86–3.50; *p* < 0.001), ever treated a patient (aRR = 3.27; 95% CI: 2.10–5.10; *p* < 0.001) and treated a patient in the last year (aRR = 2.36; 95% CI: 1.79–3.12; *p* < 0.001).

#### Catatonia rating scales

Participants from Ethiopia (aRR = 1.85; 95% CI: 1.22–2.79) and from other countries (aRR = 1.85; 95% CI: 1.10–3.10) demonstrated greater knowledge of rating scales than those from Uganda (*p* = 0.004). Psychiatry was associated with greater knowledge of rating scales (aRR = 3.34; 95% CI: 1.32–8.46; *p* = 0.005). Experience with catatonia was strongly associated with sufficient knowledge. Having ever seen a patient (aRR = 5.38; 95% CI: 1.77–16.30; *p* = 0.003), seen one in the past year (aRR = 1.94; 95% CI: 1.30–2.89; *p* = 0.001), ever treated a patient (aRR = 2.89; 95% CI: 1.66–5.04; *p* < 0.001) and treated in the past year (aRR = 1.64; 95% CI: 1.13–2.37; *p* = 0.009) were all significant predictors.

#### Catatonia complications

Female participants were more likely to correctly identify complications compared with males (aRR = 1.32; 95% CI: 1.01–1.72; *p* = 0.041). The country of practice was significantly associated with knowledge of catatonia complications. Participants from Kenya (aRR = 1.24; 95% CI: 0.80–1.93) and those in the other category (aRR = 1.53; 95% CI: 1.09–2.14) performed better than those from Uganda (*p* < 0.001). Sub-speciality was also significantly associated with knowledge of catatonia complications. Compared to neurology, all other sub-specialities demonstrated lower knowledge of catatonia complications (*p* < 0.001). More information is shown in Table 4.

**TABLE 4:** Multivariate modified Poisson regression models for factors associated with sufficient catatonia knowledge according to the sub-domains.

Factor	Level	Catatonia classification			Catatonia symptoms			Diagnostic symptoms			Catatonia screening			Catatonia rating scales			Catatonia treatment			Catatonia complications		
		aRR	95% CI	*p*	aRR	95% CI	*p*	aRR	95% CI	*p*	aRR	95% CI	*p*	aRR	95% CI	*p*	aRR	95% CI	*p*	aRR	95% CI	*p*
Gender	-	-	-	-	-	-	-	-	-	-	-	-	-	-	-	-	-	-	-	-	-	0.041
	Male	Ref.	Ref.	0.144	Ref.	Ref.	-	Ref.	Ref.	0.755	Ref.	Ref.	0.798	Ref.	Ref.	0.626	Ref.	Ref.	0.196	Ref.	Ref.	-
	Female	1.13	0.95; 1.35	-	1.08	0.79; 1.50	-	0.96	0.77; 1.20	-	1.02	0.83; 1.27	-	1.08	0.78; 1.50	-	1.18	0.91; 1.53	-	1.32	1.01; 1.72	-
Age (years)	-	-	-	-	-	-	-	-	-	-	-	-	-	-	-	-	-	-	-	-	-	0.452
	25 & below	Ref.	Ref.	0.495	Ref.	Ref.	0.460	Ref.	Ref.	0.593	Ref.	Ref.	0.586	Ref.	Ref.	0.681	Ref.	Ref.	0.759	Ref.	Ref.	-
	26–35	1.34	0.84; 2.12	-	1.60	0.72; 3.59	-	1.01	0.65; 1.59	-	1.18	0.78; 1.77	-	1.00	0.51; 1.95	-	1.06	0.63; 1.77	-	1.18	0.64; 2.17	-
	36–45	1.22	0.74; 1.99	-	1.31	0.54; 3.15	-	0.91	0.55; 1.51	-	1.01	0.62; 1.64	-	1.24	0.60; 2.58	-	1.11	0.63; 1.97	-	0.96	0.49; 1.86	-
	46–55	1.11	0.62; 1.98	-	1.77	0.67; 4.65	-	1.22	0.71; 2.07	-	1.13	0.62; 2.06	-	1.02	0.39; 2.63	-	1.39	0.74; 2.62	-	1.47	0.73; 2.96	-
	Above 55	0.97	0.42; 2.21	-	2.34	0.79; 6.89	-	0.70	0.25; 1.99	-	0.57	0.17; 1.90	-	0.40	0.05; 3.00	-	1.36	0.55; 3.31	-	1.00	0.35; 2.81	-
Country	-	-	-	-	-	-	-	-	-	-	-	-	-	-	-	-	-	-	-	-	-	0.031
	Uganda	Ref.	Ref.	< 0.001	Ref.	Ref.	0.865	Ref.	Ref.	< 0.001	Ref.	Ref.	< 0.001	Ref.	Ref.	0.004	Ref.	Ref.	0.107	Ref.	Ref.	-
	Kenya	1.35	1.05; 1.73	-	1.22	0.71; 2.10	-	1.22	0.83; 1.79	-	1.15	0.72; 1.86	-	1.13	0.61; 2.08	-	1.05	0.63; 1.77	-	1.24	0.80; 1.93	-
	Tanzania	0.91	0.70; 1.18	-	0.97	0.63; 1.49	-	0.96	0.69; 1.33	-	1.10	0.77; 1.56	-	0.87	0.52; 1.46	-	1.30	0.92; 1.83	-	0.95	0.66; 1.36	-
	Ethiopia	1.11	0.87; 1.43	-	0.83	0.50; 1.38	-	1.39	1.05; 1.85	-	2.13	1.64; 2.76	-	1.85	1.22; 2.79	-	1.47	1.03; 2.11	-	0.75	0.48; 1.18	-
	Other†	1.54	1.25; 1.90	-	1.03	0.55; 1.95	-	1.77	1.33; 2.35	-	1.85	1.31; 2.61	-	1.85	1.10; 3.10	-	1.59	1.05; 2.41	-	1.53	1.09; 2.14	-
Speciality	-	-	-	-	-	-	-	-	-	-	-	-	-	-	-	-	-	-	-	-	-	0.906
	Medicine	Ref.	Ref.	< 0.001	Ref.	Ref.	0.965	Ref.	Ref.	0.005	Ref.	Ref.	< 0.001	Ref.	Ref.	0.005	Ref.	Ref.	0.431	Ref.	Ref.	-
	Surgery	1.52	0.81; 2.85	-	1.33	0.54; 3.28	-	1.86	0.69; 4.97	-	3.41	1.43; 8.14	-	1.08	0.24; 4.84	-	1.47	0.77; 2.81	-	1.26	0.59; 2.67	-
	Obstetrics	0.97	0.35; 2.70	-	1.24	0.41; 3.74	-	2.23	0.84; 5.96	-	2.22	0.71; 6.98	-	1.77	0.41; 7.65	-	1.48	0.73; 3.01	-	1.22	0.50; 2.94	-
	Paediatrics	1.63	0.83; 3.19	-	1.53	0.54; 4.33	-	1.25	0.32; 4.84	-	-	-	-	1.71	0.40; 7.27	-	0.60	0.15; 2.30	-	0.91	0.30; 2.70	-
	Psychiatry	2.15	1.35; 3.43	-	1.28	0.64; 2.57	-	3.30	1.62; 6.69	-	4.72	2.25; 9.91	-	3.34	1.32; 8.46	-	1.30	0.78; 2.18	-	1.18	0.69; 2.02	-
	Other‡	1.18	0.83; 2.21	-	1.17	0.60; 2.29	-	2.34	1.14; 4.83	-	2.13	0.97; 4.65	-	1.47	0.55; 3.93	-	1.01	0.60; 1.69	-	1.00	0.58; 1.71	-
Sub-speciality	-	-	-	-	-	-	-	-	-	-	-	-	-	-	-	-	-	-	-	-	-	< 0.001
	Neurology (adult/paediatric)	Ref.	Ref.	0.007	Ref.	Ref.	< 0.201	Ref.	Ref.	< 0.101	Ref.	Ref.	-	Ref.	Ref.	-	Ref.	Ref.	0.338	Ref.	Ref.	-
	Neurosurgery (adult/paediatric)	0.57	0.12; 2.58	-	-	-	-	5.21	0.82; 33.12	-	-	-	-	-	-	-	0.83	0.16; 4.29	-	-	-	-
	General psychiatry	0.75	0.42; 1.35	-	1.51	0.25; 9.12	-	3.03	0.47; 19.56	-	-	-	-	-	-	-	0.60	0.29; 1.24	-	0.39	0.13; 1.17	-
	Child and Adolescent Psychiatry	0.95	0.48; 1.84	-	1.31	0.16; 10.7	-	3.77	0.55; 25.6	-	-	-	-	-	-	-	0.88	0.36; 2.14	-	0.47	0.12; 1.77	-
	Addiction psychiatry	0.43	0.15; 1.25	-	1.21	0.10; 14.7	-	1.35	0.10; 16.75	-	-	-	-	-	-	-	0.81	0.25; 2.54	-	0.75	0.23; 2.46	-
	Other psychiatry	0.58	0.29; 1.16	-	1.28	0.19; 8.66	-	3.21	0.49; 21.07	-	-	-	-	-	-	-	0.50	0.19; 1.30	-	0.42	0.12; 1.41	-
	Infectious disease (adult/paediatric)	0.67	0.34; 1.32	-	0.60	0.07; 5.09	-	1.81	0.25; 12.78	-	-	-	-	-	-	-	0.62	0.26; 1.45	-	0.24	0.06; 0.96	-
	Other§	0.51	0.28; 0.91	-	1.31	0.22; 7.66	-	2.00	0.31; 12.81	-	-	-	-	-	-	-	0.48	0.24; 0.96	-	0.39	0.13; 1.12	-
Have you ever seen a patient with catatonia?	-	-	-	-	-	-	-	-	-	-	-	-	-	-	-	-	-	-	-	-	-	0.184
	No	Ref.	Ref.	< 0.001	Ref.	Ref.	0.436	Ref.	Ref.	0.005	Ref.	Ref.	0.001	Ref.	Ref.	0.003	Ref.	Ref.	0.669	Ref.	Ref.	-
	Yes	2.36	1.51; 3.68	-	1.22	0.73; 2.01		2.01	1.23; 326	-	2.96	1.52; 5.75	-	5.38	1.77; 16.30	-	1.08	0.73; 1.60	-	1.34	0.86; 2.07	-
Have you seen catatonia within the last year?	-	-	-	-	-	-	-	-	-	-	-	-	-	-	-	-	-	-	-	-	-	0.734
	No	Ref.	Ref.	< 0.001	Ref.	Ref.	0.835	Ref.	Ref.	< 0.001	Ref.	Ref.	< 0.001	Ref.	Ref.	0.001	Ref.	Ref.	0.699	Ref.	Ref.	-
	Yes	1.50	1.23; 1.83	-	1.03	0.73; 1.47	-	1.64	1.27; 2.11	-	2.55	1.86; 3.50	-	1.94	1.30; 2.89	-	1.05	0.80; 1.39	-	1.04	0.79; 1.38	-
Have you ever treated a patient with catatonia?	No	Ref.	Ref.	< 0.001	Ref.	Ref.	0.197	Ref.	Ref.	< 0.001	Ref.	Ref.	< 0.001	Ref.	Ref.	< 0.001	Ref.	Ref.	0.321	Ref.	Ref.	0.517
	Yes	1.67	1.29; 2.17	-	0.78	0.54; 1.13	-	1.85	1.33; 2.57	-	3.27	2.10; 5.10	-	2.89	1.66; 5.04		1.17	0.85; 1.61	-	1.11	0.80; 1.53	-
Have you treated catatonia in the last year?	-	-	-	-	-	-	-	-	-	-	-	-	-	-	-	-	-	-	-	-	-	0.631
	No	Ref.	Ref.	< 0.001	Ref.	Ref.	0.359	Ref.	Ref.	0.004	Ref.	-	< 0.001	-	Ref.	0.009	Ref.	Ref.	0.063	Ref.	Ref.	-
	Yes	1.50	1.24; 1.82	-	0.84	0.59; 1.20	-	1.42	1.11; 1.81	-	2.36	1.79; 3.12	-	1.64	1.13; 2.37	-	1.30	0.98; 1.71	-	1.07	0.80; 1.41	-

aRR, adjusted risk ratio; CI, confidence interval; Ref., reference.

† , Other countries include: South Africa, the Democratic Republic of Congo and Somalia;

‡ , Other specialities include: medical officers and doctors in early training, such as interns;

§ , Other sub-specialities include: cardiology, endocrinology, nephrology and emergency medicine.

## Discussion

The study sought to examine medical practitioners’ knowledge of catatonia at participating institutions in the ACR. The number of participants with low and sufficient catatonia knowledge was almost equal. More participants had sufficient knowledge of catatonia classification and diagnostic symptoms, but low knowledge of catatonia symptoms, screening, rating scales, treatment and complications. The majority of participants had seen or treated a patient with catatonia. The country of origin, medical speciality, sub-speciality training and prior experience with catatonia (having seen or treated) were repeatedly associated with better knowledge in many sub-domains. However, none of the factors was significantly associated with knowledge of catatonia treatment.

We found that most practitioners reported having seen patients with catatonia, and a bigger proportion reported having treated them. This finding could support the evidence that catatonia is highly prevalent in our setting.^6,15^ It also builds a case for the ACR to build research capacity to better understand this debilitating condition. Medical practitioners with prior experience with catatonia had sufficient knowledge of catatonia, specifically in its classification, diagnostic symptoms, screening and rating scales. In addition, prior experience with catatonia was significantly associated with better knowledge of the above domains. This implies that grounding in knowledge is required for medical practitioners to recognise and subsequently manage catatonia. It could also imply that direct clinical experience reinforces training content, increases familiarity with disease presentations and prompts clinicians to engage with screening and rating scales. This finding supports evidence from previous studies that experiential learning is important in improving clinician competence.^16^

The majority of the participants had sufficient knowledge for catatonia classification. This differs from a previous study, in which the majority of practitioners from the United States had limited knowledge of catatonia classification.^12^ The low level of knowledge reported in previous studies may be attributed to fluctuations and changes in classification systems, as well as variations in the naming and grouping of signs across different diagnostic criteria and rating scales.^17,18^

Practitioners specialising in surgery, paediatrics, psychiatry, obstetrics and other specialities had more sufficient knowledge across classification, diagnostic symptoms, screening and rating scales than their counterparts in internal medicine. Psychiatry specialists were found to have more sufficient knowledge of catatonia, compared to non-psychiatry specialists. This is consistent with previous studies where psychiatrists performed better than other specialities.^19^ This could be attributed to differences in training traditions, leading to variations in the use of medical nomenclature and terminology.^19^ Psychiatrists utilise diagnostic criteria in the DSM-5 and ICD-11 in which catatonia is articulated as a consequence of another medical or mental disorder or an independent disease.^20,21^ Psychiatrists, therefore, encounter catatonia in their training more frequently than other specialisations. However, the low number of non-psychiatry specialists who responded to the survey suggests that these results should be interpreted cautiously. Training in any sub-speciality was significantly associated with more sufficient knowledge of classification and complications, suggesting that additional training beyond the general speciality improves knowledge.^22^

Although there were similar numbers of participants with sufficient knowledge of diagnostic symptoms, the majority had low knowledge of catatonia symptoms. The low knowledge of catatonia symptoms could be attributed to inadequate appreciation of catatonia phenotypes, as many illustrations depict it as only a hypokinetic state.^12,23^ Many practitioners may fail to recognise catatonia symptoms that constitute other catatonia phenotypes. Having sufficient knowledge of catatonia classification does not automatically imply knowing which symptoms define each specific catatonic state. This further strengthens the findings of previous studies that reported inconsistencies between theoretical and practical knowledge.^12^

The majority of the medical practitioners had low knowledge of screening and rating scales. This is because many clinicians are unclear about what constitutes each catatonic feature, how similar items should be distinguished and how specific findings should be scored on rating scales.^12,17^ The fact that only a few of the many symptoms (more than 10) on the rating scales, and different criteria, are required to diagnose catatonia contributes to the challenge, as hundreds of unique symptom combinations qualify as catatonia.^20,21,24^ In addition, many of the items on the rating scales and criteria can present in a multitude of ways that may not be recognised as catatonia (e.g., hand waving, head rocking, and foot tapping are examples of stereotypy).^12^ Some symptoms, such as staring, which are common in catatonia and are included in rating scales, are not included in the DSM-5 criteria commonly used in our setting.^6^ Medical practitioners who are trained or rely on such criteria may not recognise some symptoms of catatonia. This indicates that, although medical practitioners may have some general knowledge of catatonia, they may not appreciate its detailed symptomatology. Medical practitioners in low-resource settings may thus not be able to make a correct diagnosis or assess the severity of catatonia using different diagnostic criteria and rating scales.

There was low knowledge of the treatment of catatonia. This shows that although the majority of medical practitioners are aware of the classification of catatonia, they may fail to identify it accurately or treat it optimally. This re-emphasises observations from previous studies that patients with catatonia may receive some form of treatment that prevents the natural course of the disease.^23^ However, this does not tantamount to treatment and may not alleviate catatonia complications. This implies that patients suffering from catatonia in our setting do not receive optimal treatment and are at high risk of suffering from complications. Knowledge of catatonia treatment was not significantly associated with any parameter, indicating that even practitioners who are familiar with its identification and have prior experience with it do not necessarily have the confidence to treat it. Indeed, previous studies have shown that many practitioners incorrectly identify medication that is not recommended, such as first-generation antipsychotics, as the appropriate treatment for catatonia.^11^ This indicates a target for educational intervention about the treatment of catatonia.

There was insufficient knowledge of catatonia complications and their management. This implies that healthcare workers might fail to attribute complications to untreated catatonia. This might increase morbidity and mortality of patients who suffer from catatonia. The knowledge of complications is significantly linked with sub-speciality and country, emphasising that country-level factors and differences in medical training play an important role in catatonia knowledge.^25^

### Strengths of the study

This study documents knowledge of catatonia among medical practitioners from diverse specialities across more than five countries participating in the ACR. To the best of our knowledge, this is the first study to report on knowledge of catatonia among medical practitioners in sub-Saharan Africa, and this opens the gates for more work on catatonia to be conducted in our setting.

### Limitations

The majority of our participants were specialists in psychiatry. This shows that the respondents were more interested in catatonia and likely more knowledgeable. However, the study’s findings reveal significant knowledge gaps among both psychiatrists and non-psychiatrists.

### Recommendations

We recommend more awareness and education on catatonia among medical practitioners in low-resource settings. Speciality-specific efforts should be made in the training of specialists to aid in the prevention, early identification and management of catatonia and its complications.

## Conclusion

Medical practitioners at the partnering institutions of the ACR often encounter patients with catatonia. Although there is some knowledge of catatonia and its diagnostic criteria, there is low knowledge of its symptoms, treatment and management of complications. Since many clinicians have encountered patients with catatonia, there is a need for interventions to improve this knowledge, ultimately leading to better identification and management.
